# Integrating Patient Perspectives into Personalized Medicine in Idiopathic Pulmonary Fibrosis

**DOI:** 10.3389/fmed.2017.00226

**Published:** 2017-12-20

**Authors:** Catharina C. Moor, Peter Heukels, Mirjam Kool, Marlies S. Wijsenbeek

**Affiliations:** ^1^Department of Respiratory Medicine, Erasmus Medical Center, University Hospital Rotterdam, Rotterdam, Netherlands

**Keywords:** idiopathic pulmonary fibrosis, health-related quality of life, personalized medicine, patient-reported outcomes, personomics, patient experiences

## Abstract

Idiopathic pulmonary fibrosis (IPF) is a progressive and ultimately fatal disease which has a major impact on patients’ quality of life (QOL). Except for lung transplantation, there is no curative treatment option. Fortunately, two disease-modifying drugs that slow down disease decline were recently approved. Though this is a major step forward, these drugs do not halt or reverse the disease, nor convincingly improve health-related QOL. In daily practice, disease behavior and response to therapy greatly vary among patients. It is assumed that this is related to the multiple biological pathways and complex interactions between genetic, molecular, and environmental factors that are involved in the pathogenesis of IPF. Recently, research in IPF has therefore started to focus on developing targeted therapy through identifying genetic risk factors and biomarkers. In this rapidly evolving field of personalized medicine, patient factors such as lifestyle, comorbidities, preferences, and experiences with medication should not be overlooked. This review describes recent insights and methods on how to integrate patient perspectives into personalized medicine. Furthermore, it provides an overview of the most used patient-reported outcome measures in IPF, to facilitate choices for both researchers and clinicians when incorporating the patient voice in their research and care. To enhance truly personalized treatment in IPF, biology should be combined with patient perspectives.

## Introduction

*Give different ones [therapeutic drinks] to different patients, for the sweet ones do not benefit everyone, nor do the astringent ones, nor are all patients able to drink the same things **Hippocrates*** (1)

Idiopathic pulmonary fibrosis (IPF) is the most common idiopathic interstitial pneumonia (2). IPF is characterized by progressive decline of lung function, with a median survival of only 3–5 years (3). Common symptoms as breathlessness, cough, and fatigue have a major impact on the quality of life (QOL) of patients (4). IPF occurs more often in men than women and usually affects elderly patients, aged 50 years and above (3). There are two approved anti-fibrotic drugs that slow down disease decline, but these drugs do not halt or reverse the disease, and ultimately IPF remains a fatal disease (5, 6). The heterogeneity in disease behavior and response to therapy in IPF has (further) stimulated research to identify possible distinct underlying genetic, molecular, and environmental factors associated with IPF (7, 8).

The potential to enhance personalized treatment has prompted excitement also in the IPF field (7). Until now, the focus of personalized medicine has been on physiology and the use of this biological information to predict response to treatment and to develop targeted therapy (9). In this process, patient factors should not be overlooked. For real personalized treatment patient perceptions and preferences should also be taken into account. In this article, we focus on recent insights and methods on how to integrate patient perspectives into personalized medicine.

### Impact of Disease

Idiopathic pulmonary fibrosis is a heterogeneous disease, with a highly variable disease course (10, 11). Additionally, different phenotypes of IPF exist. Most patients have a slow disease progression, while some patients display relative stable periods followed by acute exacerbations and a small group of patients experiences a rapid decline in lung function (12). Uncertainty about the disease course and prognosis can cause emotional distress and anxiety, and, as a result, IPF has a major impact on most patients’ health-related quality of life (HRQOL). HRQOL can be defined as a patient’s perceived well-being affected by disease and treatment of the disease (13). IPF affects patients in almost every domain of life; hence, the burden of the disease is high, not just for patients but also for their partners and families. Patients often struggle with loss of independence because of functional limitations and deteriorating symptoms. Not only can breathlessness, cough, and fatigue diminish QOL, but also other symptoms such as sleep disorders, loss of appetite, and psychological problems can (14–18).

Most clinical trials in IPF that have been performed so far, have shown no convincing improvement of patient HRQOL (5, 6, 19). To date, the main focus in research has been to stabilize or improve physiological outcomes rather than HRQOL. Physiological parameters, such as lung function, do not correlate well with HRQOL measurements (20, 21). To our knowledge for parameters as imaging and biomarkers, relationships with HRQOL have not yet been established. Thus, decline in lung function does not adequately reflect the perceived impact of the disease on patients’ lives.

Every person has a different lifestyle, personal circumstances, and coping strategies. These factors can play an important role in how a disease manifests itself; hence, the same disease affects each person in a different way (16, 22, 23). Medication may show promising results at group level in randomized controlled trials, but still in some individual patients, treatment may fail (22). For example, the side effects of medication may outweigh the positive effects of medication in daily practice, or the burden of treatment might be too high for patients. To improve and personalize treatment of IPF, we should also include patient perspectives and QOL.

### Personomics

**Figure 1:**
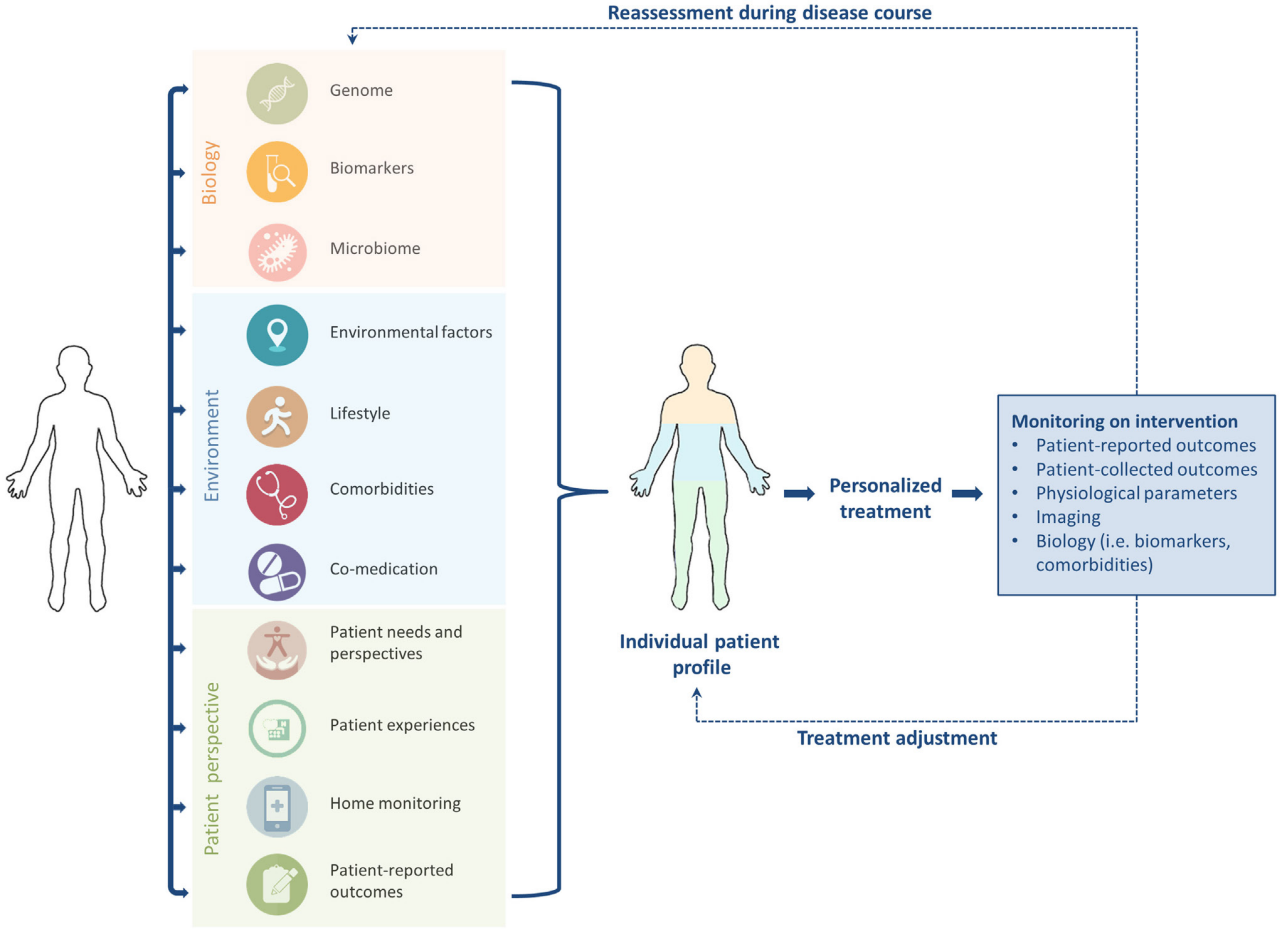
To enhance tailored treatment in idiopathic pulmonary fibrosis, “biology” should be combined with patient factors to generate an individual patient profile. Close monitoring, timely reassessment, and treatment adjustment during the disease course are required to optimize personalized care.

Personalized, stratified, or precision medicine is a broad term which can be referred to as “delivering the right treatment to the right patient at the right time” (24). Personalized medicine has gained increasing attention during the past decade (22, 25). However, the concept is not new; Hippocrates already mentioned the importance of a personalized approach to diagnosis and treatment in the fifth century BC, stating that “individuality of human beings affects predisposition to disease and response to treatment,” and also noting that “not all patients are able to drink the same therapeutic drinks” (1, 26). His concepts already include the notion that experiences with treatment differ among patients. This idea is also acknowledged by Britten et al., who suggest that because individuals are more than their genetic profile, the main concept of stratified medicine is too limited at the moment (22). Personalized treatment comprises not only “biology,” but should also focus on patient perspectives, needs, experiences, personality, environment, lifestyle, and other personal circumstances (Figure 1) (9, 22). Accordingly, the term “personomics” has been introduced to capture a patient’s life circumstances that may alter disease behavior and response to treatment (23). Below we will briefly touch on the role of biology and other aspects of personalized medicine as shown in Figure 1, but the focus will be on patient perspectives.

### Current View of Personalized Medicine in IPF

In other fields, especially oncology, personalized medicine has dramatically changed clinical practice during the last few years. Biomarkers have been used to develop targeted therapy and allocate patients to individual treatment plans (27–29).

Currently, the diagnosis of IPF is based on clinical, radiological, and pathological findings (3). The exact etiology of IPF is, however, incompletely understood. One of the proposed hypotheses is the concept of dysfunctional wound healing: repeated epithelial injury and dysfunctional regeneration possibly in combination with a dysregulated immune system normally facilitating wound healing leads to fibrogenesis and, as a consequence, excessive scarring of the lung tissue (11, 30). Epithelial injury might be caused by risk factors such as cigarette smoking, micro-aspiration of gastric content, and lead to development of IPF in susceptible individuals (11). At present, it is assumed that multiple biological pathways and complex interactions between genetic, molecular, and environmental factors are involved in the pathogenesis of IPF. Improved understanding of the pathogenesis of IPF has led to the identification of potential molecular biomarkers (7, 11, 31–33). Genome-wide association studies found genetic mutations that correlate with disease risk and possibly also disease progression (34–37); subsequently, the first examples of drug–gene interactions in IPF were found (38). To date, the value of biomarkers in IPF has not been fully clarified, and, therefore biomarkers or genetic endotyping are not yet used in clinical practice (7, 33).

Novel studies in IPF suggest that the “respiratory microbiome” is also involved in IPF pathogenesis, disease progression, and mortality (39–41). Patients with IPF have a higher bacterial burden and abundance of specific pathogens in the lung microbiome than the normal population. Furthermore, interactions have been found between specific gene expression and an altered lung microbiome in IPF, which is the first evidence for host-environmental interactions in IPF (42, 43). The lung microbiome may serve as a prognostic factor in the future, and clinical trials aimed at altering the microbiome of patients with IPF have already started (44).

A detailed description of (molecular) biology and its current role and potential in the IPF field is beyond the scope of this review.

## How to Integrate Personomics into Personalized Medicine

### Patient Needs and Perspectives in IPF Care

The importance of engaging patients in IPF care has gained increasing attention during the last several years (45). Recent qualitative studies have reported a need for better education about IPF, information about specific treatment options and palliative care, and access to specialist centers and specialist nurses. Additionally, more support for caregivers is warranted (16, 17, 46–48). These recommendations underscore the idea that not only pharmacological treatment but also non-pharmacological treatment options such as oxygen therapy, pulmonary rehabilitation, psychological support, and palliative care, are an important part of personalized management. With regard to pharmacological treatment, it is important to assess the needs and perspectives of patients before starting treatment, thereby enhancing shared decision-making. For instance, some side effects of disease-modifying drugs might have a devastating impact on one patient, but be far less bothersome to other patients (22). At the moment, over-use and under-use of medication, compliance problems, and waste of medication are not unusual in IPF (22, 49, 50). Non-adherence to medication could therefore be prevented when patients’ preferences and lifestyle are taken into account (9). Since patient preferences and needs may change because of disease progression or personal circumstances, an important aspect of disease management is iterative evaluation of the situation of individual patients (16, 46, 51). Only in this way can “holistic” personalized care be given in IPF.

### Comorbidities and Co-Medication

Holistic care also means looking further than the lungs. IPF is associated with a number of pulmonary and extra-pulmonary comorbidities, such as pulmonary hypertension, respiratory infection, cardiovascular disease, emphysema, lung cancer, diabetes mellitus, venous thromboembolism, and gastroesophageal reflux (52–56). Comorbidities are more prevalent in patients with IPF than in the normal population and have a negative influence on QOL and survival (54, 56–58). Hence, early identification and treatment of comorbid conditions have the potential to improve QOL, functional outcomes, and survival for patients with IPF (53). Kreuter et al. (54) proposed the “IPF comorbidome,” which visually displays prevalence of comorbidities and their strength of association with mortality in patients with IPF. This comorbidome could be used to predict prognosis for individual patients with IPF, and thus enhance personalized treatment.

Moreover, extra attention should be paid to the frail, elderly patients who have multiple comorbidities and functional impairment (55). As a consequence, these patients might have a higher risk of harmful side effects of disease-modifying medication and should be closely monitored during treatment. Besides, polypharmacy may play an important role in this group of patients. It is generally known that polypharmacy decreases medication compliance, increases risk of adverse drug events, and might lead to impaired functional status and cognitive impairment in elderly patients (59). Furthermore, co-medication can also interfere with disease-modifying medication, and subsequently increase side effects or reduce treatment efficacy (60). Accordingly, co-medication could play an important role in the choice of pharmacological treatment in IPF. Expected risk–benefit ratio, comorbidities, and co-medication should be taken into account before pharmacological treatment is started in individual patients.

## Measuring QOL and Monitoring Treatment Response

**Figure 2:**
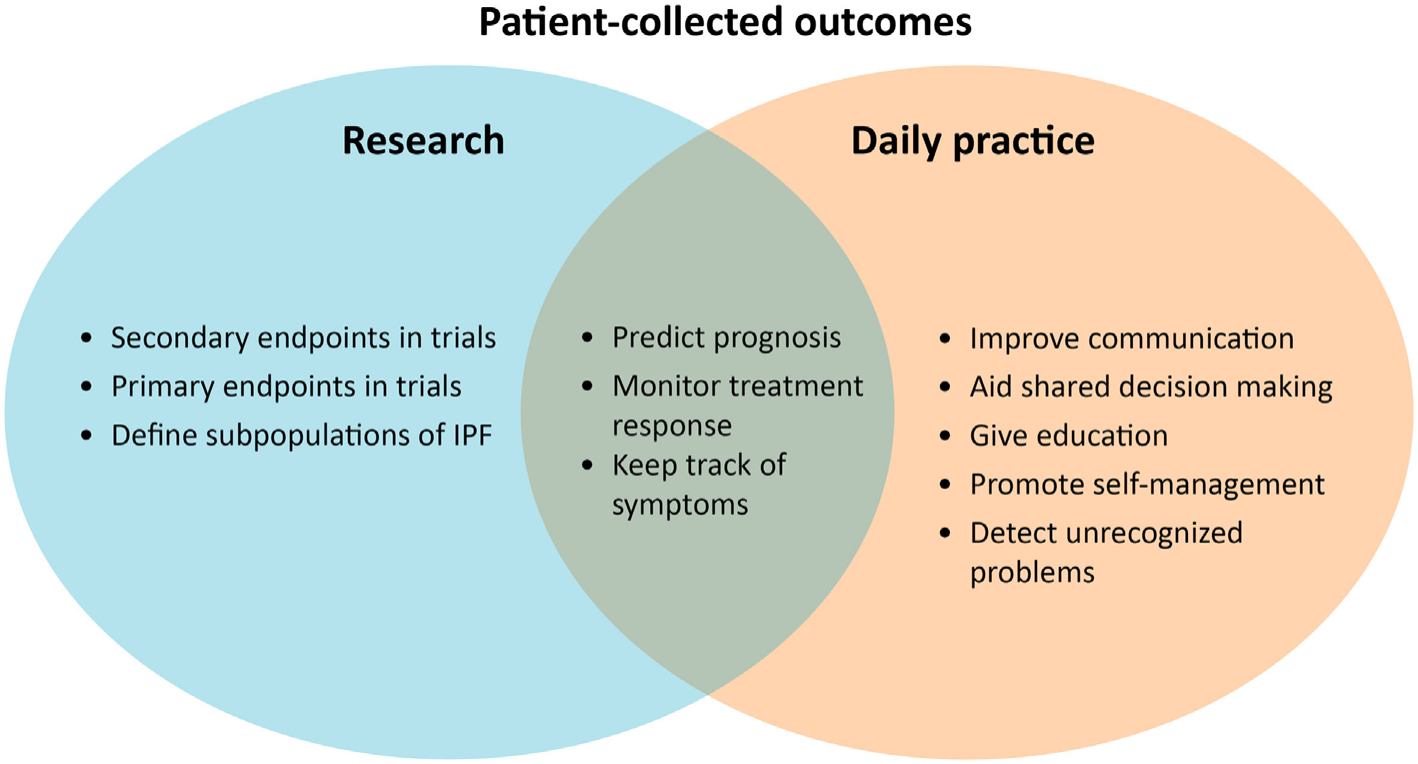
Patient-reported and recorded outcomes can be used to enhance personalized treatment.

It remains challenging how to measure patients’ disease burden, experiences, and response to treatment in IPF. For this purpose, it is important to receive structured patient input throughout the whole disease course, starting already when the diagnosis is established. At present, digital solutions can facilitate more collaboration with patients in monitoring disease behavior, their experiences, and response to therapy (Figure 2).

### Patient-Reported Outcome Measures (PROMs) in IPF

A PRO is defined as “any report of the status of a patient’s health condition that comes directly from the patient, without interpretation of the patient’s response by a clinician or anyone else” (61). Patient-reported outcome measures (PROMs) can be used to measure (HR)QOL, assess symptoms, and evaluate disease progression. There is a difference between generic and disease-specific PROMs. Disease-specific PROMs are developed to assess symptoms and (HR)QOL in a specific disease, whereas generic PROMs address more general questions and can be used in the whole population (62). One of the most commonly used generic PROMs in IPF trials are the short-form 36 and the Euroqol-5D, which is also a widely accepted instrument for economic evaluation in healthcare (63, 64). An overview of the most widely used PROMs in IPF is given in Table 1.

**Table 1:** Overview of most used patient-reported outcomes in IPF.

Patient-reported outcome measure	Description	Validation studies and MCID	Advantages	Disadvantages
**Disease-specific**				

SGRQ (65)	Fifty-item questionnaire with three domains assessing HRQOL in chronic respiratory diseases	Validated in IPF; MCID in IPF: five to eight points (66)	Used in many clinical trials in IPF	Originally developed for COPD and asthma; lengthy, difficult questionnaire

SGRQ-I (67)	IPF-specific version of original SGRQ; contains 34 items	Validity comparable with SGRQ	Questions more relevant for IPF than SGRQ	Responsiveness and MCID not known yet; limited experience

CAT (68)	Composed of eight symptom items on a 0–5 response scale	Validated in IPF	Simple and quick instrument	Originally developed for COPD; limited experience in IPF

K-BILD (21)	Fifteen-item health status questionnaire in ILD with three domains	Validated in IPF MCID in IPF: five points (69)	Brief developed in ILD including IPF patients	Limited experience in clinical trials, though increasingly used

L-IPF (70) (revised version ATAQ-IPF)	Contains two modules with different domains	Currently in validation process	Adapted with feedback from patients	Not available yet

IPF-PROM (71)	Concise questionnaire to asses QOL in IPF	Study is ongoing	Developed with patients and caregivers	Not available yet

PESaM (72)	Generic and disease-specific module; evaluates patients’ expectations, experiences, and satisfaction with disease-modifying drugs	Currently in validation process	Developed together with IPF patients	Not validated yet; responsiveness unknown

IPF-PREM (73)	Questionnaire to assess experiences with care delivery	Study is ongoing	Measures experiences of patients	Not available yet

**Domain-specific**				

UCSD (74)	Contains 24 items on a 0–5 response scale assessing dyspnea in the last week	Validated in IPF; MCID in IPF: eight points	Already used in different IPF trials; valid to assess change in dyspnea in IPF	Takes considerably more time compared with other dyspnea measures; not originally developed in IPF

mMRC (75)	Consists of one question with five grades for the level of dyspnea	Not validated in IPF	Quick, easy tool for use in daily practice; relates to disease progression	Responsiveness in IPF unclear; not originally developed in IPF

BDI-TDI (76)	BDI scores three components of dyspnea on baseline; TDI measures changes compared with baseline	Not validated in IPF; MCID in COPD: one point (76)	Measures both baseline and change over time	Only interview-administered or computerized version; not originally developed in IPF

Borg Scale (77)	Level of dyspnea scored on a scale from 0 to 10	Not validated in IPF; MCID in COPD: one point (78)	Useful during 6-min walk test in daily practice	Only measures dyspnea during exertion, does not measure dyspnea over time; not originally developed in IPF

HADS (79)	Consist of 14 items in the subscales anxiety and depression	Not validated in IPF; MCID in COPD: 1.5 points (79)	Reliable screening tool for anxiety and depression	Should not be used as diagnostic test; not originally developed in IPF

CQLQ (80)	Consists of 28 cough-specific questions in six domains	Validated in IPF; MCID in IPF: five points	Comprehensive; responsive outcome measure	Good validity for total score in IPF, but not for all domains; limited experience in IPF; not originally developed in IPF

LCQ (81)	Chronic cough quality of life questionnaire with 19 items in three domains	Not validated in IPF; MCID in chronic cough: 1.3 points (82)	High reliability; ability to detect a response to change	Limited experience in IPF; not originally developed in IPF

*IPF, idiopathic pulmonary fibrosis; MCID, minimal clinically important difference; ILD, interstitial lung disease; HRQOL, health-related quality of life; SGRQ, Saint George Respiratory Questionnaire; K-BILD, Kings’ Brief Interstitial Lung Disease health status questionnaire; L-IPF, living with idiopathic pulmonary fibrosis; ATAQ-IPF, a tool to assess quality of life in IPF; IPF-PROM, idiopathic pulmonary fibrosis—patient-reported outcome measure; PESaM, patient experiences and satisfaction with medication; IPF-PREM, idiopathic pulmonary fibrosis—patient-reported experience measure; UCSD, University of California San Diego shortness of breath; mMRC, modified Medical Research Council; BDI-TDI, baseline and transition dyspnea indexes; HADS, Hospital Anxiety and Depression Scale; CQLQ, Cough Quality of Life Questionnaire; LCQ, Leicester Cough Questionnaire*.

#### Disease-Specific PROMs

Although PROMs can play an important role to improve care for IPF, only a few well-validated, disease-specific questionnaires have been developed (19). Until a few years ago, most questionnaires used in clinical trials in IPF were originally intended for other chronic diseases (64, 65, 68). The validity of these questionnaires, such as the Saint George Respiratory Questionnaire (SGRQ) and COPD Assessment Test, has been confirmed in patients with IPF (66, 68). For the SGRQ, even an adapted version, the SGRQ-I, has been developed (67). This revised PROM consists of questions from the original SGRQ that were most relevant for patients with IPF. The reliability and validity of the SGRQ-I are comparable with the SGRQ. However, PROMs which are developed in a target population from the start, are thought to be more precise in capturing changes in HRQOL for this group of patients (58). One of the first questionnaires specifically developed in a population of patients with interstitial lung diseases (ILDs), among whom patients with IPF, is the Kings’ Brief Interstitial Lung Disease health status questionnaire (21). This is a brief, valid questionnaire that is increasingly used in IPF and other ILD clinical trials. One of the emerging PROMs in IPF is the “living with idiopathic pulmonary fibrosis” (L-IPF) questionnaire, which is a revised, electronic version of the ATAQ-IPF (a tool to assess quality of life in IPF). The L-IPF was adapted from the ATAQ-IPF following feedback from patients, and a validation study is underway at the moment (70). Another questionnaire which is currently being developed with the help of a multidisciplinary group of patients and carers is the IPF-PROM (71).

#### Domain-Specific PROMs

Additionally, domain-specific PROMs, which are questionnaires related to a specific symptom or organ, can be used to capture and objectify different aspects of disease. A few measures to evaluate breathlessness, such as the University of California San Diego Shortness of Breath Questionnaire, the modified Medical Research Council scale, the baseline and transition dyspnea indexes, and the Borg scale, are commonly used in IPF, although none were originally developed for IPF (74–77). Even though cough is a major problem in IPF, no specific cough questionnaires for IPF exist. However, the Leicester Cough Questionnaire and the Cough Quality of Life Questionnaire are currently used instead (80, 83). A widely known PROM to assess anxiety and depression is the Hospital Anxiety and Depression Scale, which is increasingly used in IPF (79). No specific fatigue questionnaires for IPF exist; however, the Fatigue Assessment Scale, originally developed for sarcoidosis, is used and might be adapted for IPF in the future (84).

### PROs in Research and Daily Practice

PROs could be very helpful to enhance personalized treatment in IPF (Figure 2). Until now, PROMs have been mainly used for research purposes, as a secondary endpoint in clinical trials. The most used primary endpoint in IPF trials is forced vital capacity, which is accepted as a surrogate measure for mortality (85). One study showed that HRQOL, assessed with the SGRQ, is also an independent prognostic factor for mortality in IPF (86). PROMs probably reflect another dimension of disease compared with traditional physiological parameters (86). In the future, PROMs could possibly be used to predict treatment success in IPF.

PROM uses in daily practice can allow healthcare providers and patients to gain more insight into the individual disease and patient behavior. In a study of Sampson et al. (46), most patients were uncertain about their own disease course and progression and had difficulties interpreting objective hospital-based parameters. PROMs could allow both patients and healthcare providers to keep track of symptoms and disease progression easily. PRO results can even be used as a simple tool to communicate with patients, educate them, promote self-management, and aid shared decision making during the course of the disease (19, 87). A systematic review in oncology has shown strong evidence that routine collecting of PROs improved patient-centered care, patient satisfaction, and detection of unrecognized problems (88).

### Patient-Reported Experience Measures in IPF

Optimal treatment requires close monitoring of the balance between the effects and side effects of disease-modifying drugs. Nonetheless, to our knowledge, a reliable measure to assess patient experiences with medication in IPF is not yet available in clinical practice. For this reason, a consortium of doctors, scientists, and patient representatives has joined forces to develop the patient experiences and satisfaction with medications (PESaM) questionnaire, which has a generic module and a disease-specific part for IPF (84). The PESaM questionnaire focuses on perceived effectiveness, side effects, and ease of use of medication and its impact on patients’ lives. This patient-reported experience measure (PREM) could not only be used in future clinical trials, but also in clinical practice to help with better detection of side effects and adjustment of medication. Moreover, Russell and colleagues, together with patients, are currently developing the “IPF-PREM.” This is a measure to assess patient experiences with healthcare and can possibly be used to improve the quality of care for patients (69).

### Home Monitoring

Ideally, for a better tailored treatment, frequent monitoring with a low burden for the patient is needed. In the last decade, the use of e-health in chronic diseases has been growing, and shows mostly promising results (89–91). E-health involves the exchange of data between a patient and a healthcare provider using information and communication technologies (92). By using e-health tools, patients may better understand their health condition and become actively involved in the management of their own disease. It allows frequent monitoring in between regular visits and collection of PROs at home (93). Recently, a study showed that daily home spirometry in a population of patients with IPF was highly feasible and informative (94). Home-based spirometry predicts disease decline and mortality better than hospital-based measurements. Routine home spirometry could be very helpful to identify patients with rapid decline in lung function and to evaluate response to treatment. The authors suggest that daily home spirometry will allow for more individualized patient care. The feasibility of home-based spirometry in IPF was confirmed by Johannson et al. (95), who additionally showed that home spirometry might reduce sample size as well as the length of future clinical trials. Another promising example of home monitoring in IPF is the longitudinal follow-up of physical activity with activity trackers worn by patients at home (96). Decline in physical activity can provide reliable, objective data on disease progression and could be integrated into a home monitoring program. A comprehensive home monitoring program, consisting of an e-health tool combined with home spirometry and online collecting of PROs, has the potential to enhance trial design, stimulate self-management, allow for early treatment adaption to minimize side effects, prevent hospital admissions, and subsequently improve personalized management and QOL for patients with IPF.

## Conclusion

The potential to enhance personalized treatment has prompted excitement also in the IPF field. In the future, patients’ genetic, biomarker, and microbiome profiles may guide clinical trial design and treatment decisions. In this process, patient perspectives should not be overlooked. Only by integrating biological information with patient-reported and patient-collected information, will we be able to realize truly personalized treatment.

## Author Contributions

All authors conceptualized and designed the review, CM and MW wrote the paper, PH and MK provided critical feedback and input. All authors agree to be accountable for the content of the work and approved the manuscript.

## Conflict of Interest Statement

The authors declare that the research was conducted in the absence of any commercial or financial relationships that could be construed as a potential conflict of interest.
